# Nutrition and psoriasis: is there any association between the severity of the disease and adherence to the Mediterranean diet?

**DOI:** 10.1186/s12967-014-0372-1

**Published:** 2015-01-27

**Authors:** Luigi Barrea, Nicola Balato, Carolina Di Somma, Paolo Emidio Macchia, Maddalena Napolitano, Maria Cristina Savanelli, Katherine Esposito, Annamaria Colao, Silvia Savastano

**Affiliations:** I.O.S. & COLEMAN Srl, Naples, Italy; Dipartimento di Medicina Clinica e Chirurgia, Unit of Dermatology, Federico II University Medical School of Naples, Via Sergio Pansini 5, Naples, 80131 Italy; IRCCS SDN, Napoli Via Gianturco 113, Naples, 80143 Italy; Department of Clinical and Experimental Medicine, Second University of Naples, Via Costantinopoli, Naples, 84-80138 Italy; Dipartimento di Medicina Clinica e Chirurgia, Unit of Endocrinology, Federico II University Medical School of Naples, Via Sergio Pansini 5, Naples, 80131 Italy

**Keywords:** Nutrition, Psoriasis, Mediterranean diet, Body composition

## Abstract

**Background:**

Many studies have evaluated the role of individual nutrients on the development of psoriasis. However, only few studies have investigated the effect of a healthy eating pattern, such as the Mediterranean diet. In this study, we aimed to investigate the relationship between adherence to the Mediterranean diet, the body composition and the severity of psoriasis in a group of naïve-treatment patients with psoriasis.

**Methods:**

This is a cross-sectional case–control observational study. Sixty-two patients (49 males and 13 females, mean age: 50.2±10.5yrs) affected with mild-to-severe psoriasis were consecutively enrolled. Sixty-two age-, sex- and body mass index (BMI)-matched healthy subjects served as control group. A validated 14-item questionnaire (PREDIMED: PREvención con DIeta MEDiterránea) was used for the assessment of adherence to the Mediterranean diet. The severity of psoriasis was by assessed by standardized Psoriasis Area and Severity Index (PASI) score and C-reactive protein (CRP) levels. Body composition was analyzed with bioelectrical impedance analysis.

**Results:**

A higher percentage of psoriatic patients had a lower PREDIMED score compared to the control group (30.6% *vs* 4.8%). PASI score was significantly associated with the percentage of fat mass (FM%) and CRP levels. PASI score and CRP levels were significantly associated with the dietary components included in the PREDIMED questionnaire or with the PREDIMED score. At multiple regression analysis, the major predictor of PASI score were FM among BIA parameters, (r^2^=0.537, β=0.740, p<0.001), and FM (r^2^=0.537, β=0.603, p<0.001) and PREDIMED score (r^2^=0.599, β=−0.296, p=0.007) among anthropometric measures, FM and PREDIMED score. Finally, among all items of the PREDIMED questionnaire, EVOO (r^2^=0.548, β=−0.741, p<0.001), and fish consumption (r^2^=0.139, β=−0.372, p=0.005) have an independent predictive value for PASI score and CRP levels.

**Conclusions:**

This is the first study to evaluate the association between adherence to the Mediterranean diet and the severity of psoriasis. Moreover, our study highlights the usefulness of the assessment of body composition by bioelectrical impedance analysis in the evaluation of the psoriatic patients.

## Background

Psoriasis is a chronic, immune-mediated inflammatory skin disease, associated with metabolic and cardiovascular disease [1,2]. Psoriasis is estimated to affect about 2–4% of the population in Western countries [3]. A number of risk factors have been recognized in the etiology and pathogenesis of psoriasis, including family history and environmental risk factors, such as diet, obesity, smoking, stress, and alcohol consumption [4]. Moreover, dietary factors can also affect both drug pharmacokinetics and pharmacodynamics. A number of single food components have been suggested to play a role in psoriasis. The ability of dietary of antioxidants, such as omega-3 polyunsaturated fatty acids from fish oil, some vitamins (A, E and C), and oligoelements (iron, copper, manganese, zinc and selenium), which decrease oxidative stress and the production of reactive oxygen species, might be of particular relevance mainly in a chronic systemic inflammatory diseases, like psoriasis [5]. In addition, due to its role in proliferation and maturation of keratinocytes, vitamin D has become an important therapeutic option in the treatment of psoriasis [6]. However, while many studies have evaluated the role of individual nutrients on the development of psoriatic disease [7], only few studies have investigated the effect of a healthful eating pattern. The psoriatic patients, in fact, often seek dietary advice, as they frequently link many of their health problems, including diseases of the skin, to their diet.

The Mediterranean diet is a healthy eating pattern, associated with reduced risk for metabolic [8], cardiovascular [9], and neoplastic diseases [10], that has consistently been shown to provide a degree of protection against chronic degenerative diseases [11]. One of the most accredited hypothesis of this association is that the high content of different beneficial compounds, such as antioxidants and polyphenols, largely present in Mediterranean foods, such as plant foods, fruits and red wine, have anti-inflammatory properties [12]. In particular, the monounsaturated fatty acids intake, whose major source is represented by extra virgin olive oil (EVOO), was found to be associated with a reduced prevalence of risk factors for major chronic inflammatory diseases [13].

Obesity is an important risk factor for psoriasis [14]. The relationship between the two conditions is probably bidirectional, with obesity predisposing to psoriasis and psoriasis favoring obesity [15]. Several studies have been conducted to explore the association between obesity and psoriasis [16,17]. In particular, there was a 2-fold increased risk for psoriasis development in the setting of obesity as compared with normal weight subjects [18]. In addition, for each unit increment increase in body mass index (BMI) was reported a 9% higher risk for psoriasis onset and a 7% higher risk for increased of Psoriasis Area and Severity Index (PASI) score [19], currently the preferred method for the assessment of the disease severity and extent. A further evidence of a link between, obesity, inflammation and cardiovascular diseases in patients with psoriasis is provided by several studies reporting a correlation between PASI score and increased of C-reactive protein (CRP) levels [20], an acute phase protein significantly associated with obesity, representing the most sensitive markers of inflammation and an independent risk for cardiovascular disease. BMI is a commonly used surrogate for adiposity that is inexpensive and easily measured, although it evaluates excess weight rather than excess fat [21], does not measure body fat directly and poorly distinguishes between fat mass and lean or bone mass [22]. The National Heart, Lung and Blood Institute (NHLBI) Clinical Guidelines recognize this limitation of BMI [23]; thus, waist circumference (WC) is recommended as an additional surrogate measure of fat distribution, due to its high correlation with visceral fat [24], the main source of inflammatory cytokines in obesity [25]. Again, a number of studies have evidenced a strict association between WC and psoriasis [26], being the production of inflammatory cytokines in visceral obesity the link involved in the complex mechanisms leading to the exacerbation of psoriasis [27]. Nevertheless, only few studies have assessed body composition in psoriatic patients [28,29]. Bioelectrical impedance analysis (BIA) is a simple, safe, inexpensive and non-invasive method to estimate body composition. This method is particularly useful in determining body compartments in studies of large population samples [30].

The wake of this evidence, we aimed to investigate the relationship between adherence to the Mediterranean diet using a 14-item PREDIMED (PREvención con DIeta MEDiterránea) questionnaire [31], the body composition by BIA, and the severity of psoriasis evaluated by PASI score and CRP levels in a group of naïve-treatment patients with psoriasis.

## Methods

This is a cross-sectional case control observational study carried out at the Department of Clinical Medicine and Surgery of the University of Naples Federico II (Italy), in accordance with the guidelines of the Helsinki Declaration on human experimentation. All participants gave their informed consent before enrolment.

Sixty-two patients affected by mild-to-severe psoriasis were consecutively enrolled from a pool of patients referred to the outpatient clinic of our University Hospital from January to August 2014. Patients aged more than 18 years and with a diagnosis of mild-to-severe psoriasis lasting for at least 6 months were enrolled, while patients with pustular, erythrodermic or arthropathic psoriasis or receiving any systemic treatment for psoriasis including acitretin, ciclosporin, methotrexate, phototherapy or biologics for at least 3 months were excluded. Exclusion criteria included smoking and alcohol abuse too. Furthermore they had not received any drug therapy known to affect carbohydrate or lipid metabolism for the 6 months before. All patients were checked and recorded. Data collected included age, gender, weight, height, BMI and WC. Severity of psoriasis and the age at onset of it (early psoriasis: age < 39 years and late psoriasis aged >40 years) were also noted. PASI score was used as measure of disease severity. PASI score was used to be recorded by measuring four body surface areas (head, chest, upper and lower limbs) with patient in a standing position according to the method described by Harari M et al. 2000 [32]. Sixty-two healthy subjects among clerks, and medical and paramedical personnel of the Department of Clinical & Surgical Medicine, Unit of Endocrinology of the University Federico II of Naples, matched for sex, age, and BMI with the patients agreed to participate in this study and were used as controls. Exclusion criteria for controls were the same as the patients. Blood samples were obtained between 08.00 h and 09.00 h from an antecubital vein after an overnight fast, with the patient in the resting position. CRP levels was determined with a nephelometric assay with CardioPhase high-sensitivity from Siemens Healthcare Diagnostics (Marburg, Germany); the intra-assay coefficient of variations was >4%.

All anthropometric measurements were taken with subjects wearing only light clothes and without shoes. In each subject, weight and height were measured to calculate the BMI [weight (kg) divided by height squared (m^2^), kg/m^2^]. Height was measured to the nearest 1 cm using a wall-mounted stadiometer. Body weight was determined to the nearest 50 g using a calibrated balance beam scale. The degree of obesity was established according to a scale based on BMI cut-off points: 30–34.9 (grade I obesity), 36–39.9 (grade II obesity) and ≥ 40 kg/m^2^ (grade III obesity or severe obesity), respectively.

WC was measured to the closest 0.1 cm with a non-extensible tape at the natural indentation or at a midway level between the iliac crest and the lower edge of the rib cage if no natural indentation was visible. The measurement was made with the subject standing upright, feet together and arms hanging freely at the sides.

In addition, for each patient we performed body composition with bioelectrical impedance analysis (single-frequency 50 kHz BIA 101 RJL, Akern Bioresearch, Firenze) and biavector analysis, with resistance (R) and reactance (Xc) data plotted on an R/H Xc/H graph using a specific software [33]. All participants were supine with limbs slightly spread apart from the body, refrained from eating, drinking, and exercising for six hours before testing. Subjects were tested in the supine position with arms and legs abducted from the body. Shoes and socks were removed and contact areas were scrubbed with alcohol immediately before electrode placement. Electrodes (BIATRODES Akern Srl; Florence, Italy) were placed proximal to the phalangeal–metacarpal joint on the dorsal surface of the right hand and distal to the transverse arch on the superior surface of the right foot. Sensor electrodes were placed at the midpoint between the distal prominence of the radius and ulna of the right wrist, and between the medial and lateral malleoli of the right ankle.

A validated 14-item questionnaire for the assessment of adherence to the Mediterranean Diet (PREDIMED) [31] was recorded for all the enrolled subjects during a face-to-face interview between the patient and a certified nutritionist. Briefly, for each items was assigned score 1 and 0; PREDIMED score was calculated as follows: 0–5, lowest adherence; score 6–9, average adherence; score ≥10, highest adherence [31].

Results are expressed as mean ± SD. Differences between the two groups were analyzed by paired *t* test. Frequencies were analyzed by *χ*^2^ test. Correlations between variables were performed using Pearson *r* correlation coefficient. Using PASI score and CRP levels as dependent variables, three multiple linear regression analysis models (stepwise method) were set up to estimate the predictive value of: a) body composition evaluated by BIA, b) BMI, WC, FM and PREDIMED score; c) each item included in PREDIMED questionnaire (expressed as r^2^, Beta (β) and t). In these analysis, we entered only those variables that had a p-value <0.05 in the univariate analysis. To avoid multicollinearity, variables with a variance inflation factor (VIP) >10 were excluded. Values ≤5% were considered statistically significant. Data were stored and analysed using the MedCalc® package (Version 12.3.0 1993–2012 MedCalc Software bvba - MedCalc Software, Mariakerke, Belgium).

## Results

**Figure 1:**
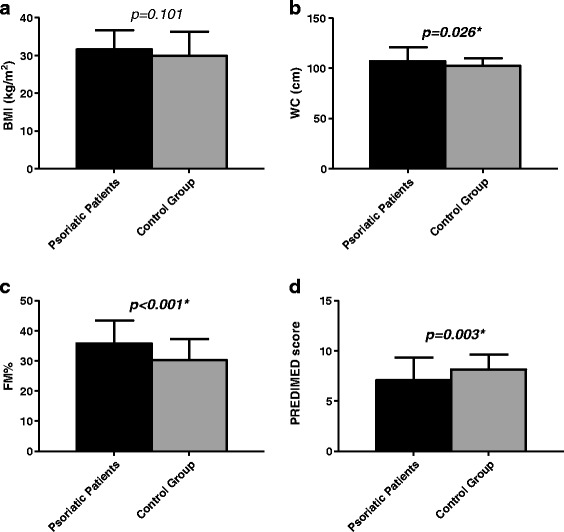
**Anthropometric and nutritional differences between psoriatic patients and control group.** In psoriatic patients BMI **(a)**, WC **(b)** and FM **(c)** were significantly higher and PREDIMED score **(d)** was lower than in control group**.** Results are expressed as mean ± SD. Differences between groups were analyzed by paired *t* test. **BMI**, body mass index; **WC**, waist circumference; **FM**, Fat Mass; **PREDIMED**, PREvención con DIeta MEDiterránea.

**Figure 2:**
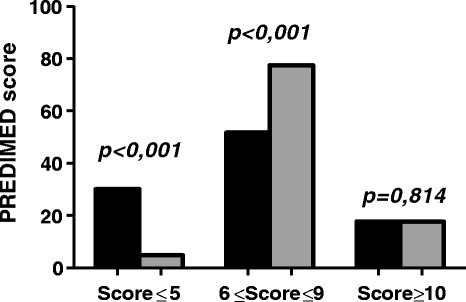
**PREDIMED score of all participants.** A higher percentage of psoriatic patients had a lower PREDIMED score compared with the control group (30.6% *vs* 4.8%). Frequencies were analyzed by *χ* ^2^ test. Score 0–5, lowest adherence to the Mediterranean diet; Score 6–9, average adherence to the Mediterranean diet; Score ≥10, highest adherence to the Mediterranean Diet. **PREDIMED**, PREvención con DIeta MEDiterránea.

A total of 62 subjects (49 males and 13 females) for group completed the study. Among psoriatic patients PASI score was 7.9 ± 7.2 and CRP levels were 2.4 ± 5.4 ng/ml (control group: 0.7 ± 0.4 ng/ml; p = 0.040). In Table 1 are shown the differences in the anthropometric and BIA parameters of the psoriatic patients and the control group. No statistically significant differences in age was and BMI were observed between the two groups. Psoriatic patients exhibited statistically significant differences compared with control group for BIA parameters. In particular psoriatic patients have the lowest values of Na/K ratio, FFM (%), SMM (%), TBW (%), and higher values of FM (Kg) and FM (%). Moreover, in psoriatic patients WC were and FM were significantly higher and PREDIMED score was lower than in control group; Figure 1. In Table 2 are reported the responses of each item included in PREDIMED questionnaire in the two groups. Psoriatic patients exhibited statistically significant differences compared with controls for use of the following dietary components: EVOO, fruits, red processed meats, fish and nuts. The total PREDIMED score in the two groups is reported in Figure 2. A higher percentage of psoriatic patients had a lower/average adherence score compared to the control group (30.6% *vs* 4.8%, p < 0.001 and 51.7% *vs* 77.5%, p = 0.004, respectively) as assessed by the PREDIMED questionnaire, while there no significant differences with the high adherence score (17.7% *vs* 17.7%, p = 0.814).

**Table 1:** **Anthropometric measures and body composition evaluated by BIA parameters of the psoriatic patients and control group**

	**Psoriatic patients**	**Control group**	
	***Mean ± SD***	***Mean ± SD***	***p*** **value**
Age (years)	50.2 ± 10.5	49.8 ± 10.4	0.857
BW (kg)	88.3 ± 15.1	83.0 ± 17.0	0.069
Height (m)	1.7 ± 0.1	1.7 ± 0.1	0.747
BMI (kg/m^2^)	31.6 ± 5.1	29.9 ± 6.5	0.101
R (Ω)	556.3 ± 116.6	526.4 ± 79.7	0.087
Xc (Ω)	43.5 ± 6.7	42.5 ± 6.5	0.453
PA (°)	4.6 ± 1.0	4.7 ± 1.4	0.807
R/H (Ω/m)	313.7 ± 99.2	315.6 ± 51.4	0.895
Xc/H (Ω/m)	24.6 ± 7.1	25.8 ± 3.9	0.291
Na/K ratio	1.1 ± 0.2	1.2 ± 0.2	**0.032**
BCMI	7.4 ± 4.0	7.6 ± 3.0	0.702
FM (kg)	32.0 ± 9.5	25.4 ± 9.7	**0.001**
FM (%)	35.6 ± 6.8	30.3 ± 6.6	**<0.001**
FFM (kg)	57.3 ± 10.3	56.4 ± 9.4	0.605
FFM (%)	64.4 ± 6.8	69.5 ± 6.8	**<0.001**
BCM (kg)	20.6 ± 11.2	20.9 ± 8.3	0.824
BCM (%)	34.8 ± 12.1	36.7 ± 9.3	0.288
SMM (kg)	26.7 ± 12.7	27.0 ± 9.5	0.854
SMM (%)	29.7 ± 11.2	33.2 ± 8.7	**0.042**
TBW (Lt)	41.9 ± 7.6	41.3 ± 6.9	0.602
TBW (%)	47.2 ± 4.9	51.1 ± 4.8	**<0.001**
TBW/BW	0.48 ± 0.06	0.51 ± 0.06	**0.005**
ECW (Lt)	22.2 ± 3.2	21.7 ± 2.9	0.383
ECW (%)	53.8 ± 7.1	53.2 ± 5.9	0.572
ICW (Lt)	19.7 ± 6.4	19.6 ± 5.3	0.888
ICW (%)	46.2 ± 7.1	46.9 ± 5.9	0.503

Psoriatic patients exhibited statistically significant differences compared with control group sex, age and BMI-matched for BIA parameters. In particular psoriatic patients have the lowest values of Na/K ratio, FFM (%), SMM (%),TBW (%) and higher values of FM (Kg) and FM (%). Differences between the two groups were analyzed by paired *t* test. **BW,** Body weight; **BMI,** Body Mass Index; **R,** Resistance (Ω = ohm); **Xc,** Reactance (Ω = ohm); **PA,** Phase Angle (° = degrees); **R/H,** Resistance standardized by Height (Ω/m = ohm/meters); **Xc/H,** Reactance standardized by Height (Ω/m = ohm/meters); **Na/K,** Sodium/Potassium; **BCMI,** Body Cell Mass Index; **FM,** Fat Mass; **FFM,** Fat-Free Mass; **BCM,** Body Cell Mass; **SMM** Skeletal Muscle Mass; **TBW,** Total Body Water; **ECW,** Extra-Cellular Water; **ICW,** Intra-Cellular Water.

**Table 2:** **Response frequency of dietary components included in the PREDIMED questionnaire of the psoriatic patients and control group**

	**Questions**	**Psoriatic**		**Control**		
		**Patients**		**Group**		
		**n**	**%**	**n**	**%**	***p*** **values**
1	Use of extra virgin olive oil as main culinary lipid	43	69.4	59	95.2	**<0.001**
2	Extra virgin olive oil >4 tablespoons	39	62.9	34	54.8	0.465
3	Vegetables ≥2 servings/day	36	58.1	39	62.9	0.713
4	Fruits ≥3 servings/day	45	72.6	55	88.7	**0.041**
5	Red/processed meats <1/day	29	46.8	11	17.7	**0.001**
6	Butter, cream, margarine <1/day	18	29.0	21	33.9	0.699
7	Soda drinks <1/day	24	38.7	28	45.2	0.585
8	Wine glasses ≥7/week	23	37.1	33	53.2	0.104
9	Legumes ≥3/week	39	62.9	44	71.0	0.445
10	Fish/seafood ≥3/week	32	51.6	46	74.2	**0.016**
11	Commercial sweets and confectionery ≤2/week	18	29.0	26	41.9	0.189
12	Tree nuts ≥3/week	23	37.1	35	56.5	**0.048**
13	Poultry more than red meats	31	50.0	31	50.0	0.858
14	Use of sofrito sauce ≥2/week	38	61.3	42	67.7	0.573

Psoriatic patients showed statistically significant differences compared with controls for use of the following dietary components: Extra virgin olive oil, fruits, red processed meats, fish and tree nuts. Frequencies were analyzed by *χ* ^2^ test. **PREDIMED,** PREvención con DIeta MEDiterránea.

### Correlation studies

**Figure 3:**
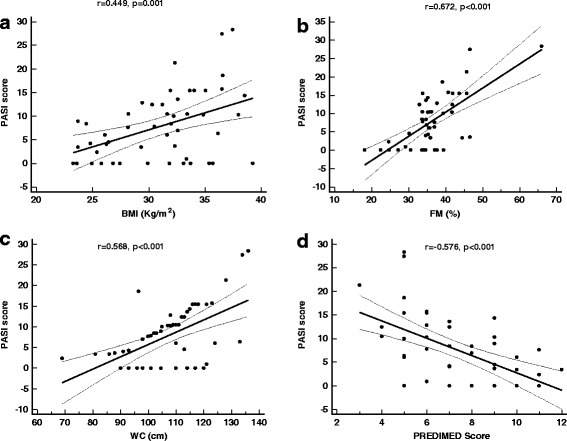
**Correlation among PASI score and anthropometric parameters, body composition and PREDIMED score.** There was a significant positive association among PASI score and BMI **(a)**, FM **(b)**, WC **(c)**, and PREDIMED score **(d).** Correlations among variables were analyzed using Pearson *r* correlation coefficient. **BMI**, body mass index; **WC**, waist circumference; **FM**, Fat Mass; **PREDIMED**, PREvención con DIeta MEDiterránea.

The correlations among PASI score, CRP levels and BIA parameters were reported in Table 3. In particular, PASI score was negatively correlated with Xc, PA, BCMI, FFM%, BCM (Kg and%), SMM (Kg and%), TBW%, TBW/BW, and ICW%. Moreover, there was a significant positive association among PASI score and BMI, FM, WC and PREDIMED score (Figure 3). The correlations between PASI score and CRP levels with each item of the PREDIMED questionnaire are showed in Table 4. At multiple regression analysis, the major predictor of PASI score were FM among BIA parameters, (r^2^ = 0.537, β = 0.740, t = 7.457, p < 0.001), and FM (r^2^ = 0.537, β = 0.603, t = 5.790, p < 0.001) and PREDIMED score (r^2^ = 0.599, β = −0.296, t = −2.839, p = 0.007) among anthropometric measures. Finally, among all items of the PREDIMED questionnaire, EVOO (r^2^ = 0.548, β = −0.741, t = −7.636, p < 0.001), and fish consumption (r^2^ = 0.139, β = −0.372, t = −2.922, p = 0.005) have an independent predictive value for PASI score and CRP levels, respectively.

**Table 3:** **Correlations among body composition evaluated by BIA, PASI score and CRP levels in psoriatic patients**

	**PASI score**		**CRP levels**	
	**r**	***p*** **values**	**r**	***p*** **values**
R (Ω)	0.131	0.375	0.093	0.529
Xc (Ω)	−0.427	**0.002**	−0.345	**0.016**
PA (°)	−0.425	**0.003**	−0.264	0.070
R/H (Ω/m)	0.153	0.288	0.115	0.422
Xc/H (Ω/m)	−0.184	0.201	−0.127	0.373
Na/K ratio	0.011	0.941	0.232	0.112
BCMI	−0.331	**0.022**	−0.191	0.193
FM (Kg)	0.740	**<0.001**	0.150	0.307
FM (%)	0.652	**<0.001**	0.242	0.098
FFM (Kg)	0.052	0.725	−0.125	0.399
FFM (%)	−0.652	**<0.001**	−0.242	0.098
BCM (Kg)	−0.342	**0.017**	−0.201	0.170
BCM (%)	−0.423	**0.003**	−0.250	0.086
SMM (Kg)	−0.322	**0.026**	−0.196	0.181
SMM (%)	−0.509	**<0.001**	−0.251	0.085
TBW (Lt)	0.051	0.732	−0.125	0.397
TBW (%)	−0.652	**<0.001**	−0.242	0.098
ECW (Lt)	0.467	**0.001**	0.158	0.284
ECW (%)	0.408	**0.004**	−0.029	0.847
ICW (Lt)	−0.228	0.119	−0.213	0.146
ICW (%)	−0.408	**0.004**	−0.303	**0.037**

Correlation among BIA parameters, PASI score and CRP levels were performed using Pearson *r* correlation coefficient. **BIA,** Bioelectrical impedance analysis**; PASI,** Psoriasis Area Severity Index; **CRP**, C-reactive protein**; R,** Resistance (Ω = ohm); **Xc,** Reactance (Ω = ohm); **PA,** Phase Angle (° = degrees); **R/H,** Resistance standardized by Height (Ω/m = ohm/meters); **Xc/H,** Reactance standardized by Height (Ω/m = ohm/meters); **Na/K,** Sodium/Potassium; **BCMI,** Body Cell Mass Index; **FM,** Fat Mass; **FFM,** Fat-Free Mass; **BCM,** Body Cell Mass; **SMM** Skeletal Muscle Mass; **TBW,** Total Body Water; **ECW,** Extra-Cellular Water; **ICW,**Intra-Cellular Water.

**Table 4:** **Correlations between PASI score and CRP levels with dietary components included in the PREDIMED questionnaire in the psoriatic patients**

	**Questions**	**PASI score**		**CRP levels**	
		**r**	***p*** **values**	**r**	***p*** **values**
1	Use of extra virgin olive oil as main culinary lipid	−0.741	**<0.001**	−0.300	**0.026**
2	Extra virgin olive oil >4 tablespoons	0.232	0.106	0.101	0.462
3	Vegetables ≥2 servings/day	−0.600	**<0.001**	−0.212	0.120
4	Fruits ≥3 servings/day	−0.628	**<0.001**	−0.350	**0.009**
5	Red/processed meats <1/day	0.427	**0.002**	0.282	**0.037**
6	Butter, cream, margarine <1/day	0.176	0.222	−0.106	0.441
7	Soda drinks <1/day	0.162	0.262	0.210	0.123
8	Wine glasses ≥7/week	−0.217	0.131	−0.145	0.290
9	Legumes ≥3/week	−0.421	**0.002**	−0.299	**0.027**
10	Fish/seafood ≥3/week	−0.618	**<0.001**	−0.372	**0.005**
11	Commercial sweets and confectionery ≤2/week	0.178	0.215	−0.007	0.959
12	Tree nuts ≥3/week	−0.365	**0.009**	−0.354	**0.008**
13	Poultry more than red meats	0.107	0.461	−0.020	0.884
14	Use of sofrito sauce ≥2/week	−0.368	**0.009**	−0.062	0.654
	***PREDIMED score***	−0.576	**<0.001**	−0.348	**0.009**

Correlations among variables were performed using Pearson *r* correlation coefficient. **PASI,** Psoriasis Area Severity Index; **CRP**, C-reactive protein; **PREDIMED,** PREvención con DIeta MEDiterránea.

## Discussion

Psoriasis is a chronic, immune-mediated inflammatory skin disease. Beyond the skin, psoriasis is associated with a systemic inflammatory state that has been linked to obesity, cardiovascular diseases, and type 2 diabetes. The causes of psoriasis are not fully understood, but a number of risk factors are recognized, including family history and environmental risk factors, such as diet, smoking, stress, obesity, and alcohol consumption [34].

The novel finding of this study is the association between the severity of psoriasis, assessed by PASI score and CRP levels, and the degree of adherence to the Mediterranean diet. In particular, the results of our study show that a higher percentage of psoriatic patients have a low adherence to the Mediterranean diet compared with the age- sex- and BMI-matched control group. In line with the current literature, we found that BMI and abdominal obesity, as measured by WC, were associated with psoriasis [26]. In addition, as also very recently reported [35] in a series of psoriatic patients (treated and untreated) compared with normal weight controls, we found that psoriatic patients exhibited statistically significant differences in body composition evaluated by BIA also compared with BMI-matched control group. In particular, we found that psoriatic patients have lower values of the ratio of TBW to body weight, greater amounts of adiposity and lower muscle mass compared with the obese counterpart without psoriasis. Although in obese patients, subtle variations of the hydration of soft tissues can propagate errors in BIA measures, BIA shows a good agreement with Dual-energy X-ray absorptiometry (DXA) [36], and the role of BIA to estimate body water and for predicting body cell mass is largely demonstrated [37]. These findings might suggest the presence of a form of sarcopenic obesity in psoriatic patients, likely linked to their chronic inflammatory disease, and let us also to hypothesize a worsening effect of psoriasis on body composition among equally obese individuals. Furthermore, after including all BIA parameters as predictive variables in the multiple regression model, FM remained the major predictor of the severity of the psoriasis. Thus, the usefulness of a careful assessment of body composition in the evaluation of the psoriatic patients of both genders is conceivable suggested.

Single food components have been suggested to play a role in aetiology and pathogenesis of psoriasis. Previous studies of the relationship of diet and nutrition with psoriasis have focused on either individual nutrients (eg, fish oil, omega 3, vitamin B_12_, vitamin D, vitamin A, selenium, inositol and zinc and antioxidants) or individual food groups (eg, fruit, vegetables, and fish) [5]. However, diet is a complex combination of foods from various groups and nutrients, and some nutrients are highly correlated. Thus, it is challenging to separate the effect of a single nutrient or food group from that of others in free-living populations [38].

One of the most frequently asked questions by patients with psoriasis is whether dietary changes could improve their disease. Given the negative impact of psoriasis on quality of life, patients often seek information about dietary and lifestyle modifications to assist in clearing their skin lesions [18]. Modifying diet is an accessible and self-empowering method that many patients are eager to embrace in treating their disease. However, despite the fact that nutrition might be considered an adjunctive tool for the treatment of psoriasis, there are no national or international guidelines that recommend an adequate diet for such patients [39]. With increasing awareness that psoriasis is associated with cardiovascular disease and metabolic syndrome, patients may also seek to improve diet to prevent these comorbidities. To the best of our knowledge, this is the first study to look at the association between adherence Mediterranean diet and psoriasis. In particular, the psoriatic patients consumed less EVOO, fruit, fish and nuts, while consumed more red meat compared to the control group.

The Mediterranean diet it is characterized by a high intake of fruit and vegetables, legumes, grains and cereals, fish and seafood and nuts; a low intake of dairy products, meat and meat products; and a moderate ethanol intake mainly in the form of wine and during meals [40]. EVOO is the main added lipid and its increased consumption is reflected in the high monounsaturated to saturated fatty acid intake [41]. The most interesting aspect of this study is that adherence to the Mediterranean diet is appraised using only a brief 14-item questionnaire (PREDIMED questionnaire) [31] which is less time-demanding, less expensive and requires less collaboration from participants than the usual full-length food frequency questionnaire (FFQ) or other more comprehensive methods [12]. Moreover, this questionnaire allows to provide feedback to the participant immediately after the interview is completed. In fact, this 14-item tool is a key element in the intervention conducted in the PREDIMED trial and has been previously validated against the FFQ used in the study [42]. In addition, we reported the specific association of each of the 14 individual items included in the PREDIMED score with the PASI score and CRP levels. In particular, PASI score and CRP levels were both negatively correlated not only with the PREDIMED score, but also the consumption of EVOO, vegetables, fruit, legumes, fish and nuts, and positively correlated with the consumption of red meat. Among these food components, EVOO and fish were independent predictors of PASI score and CRP levels, respectively. EVOO is one of many components of the Mediterranean diet. The decarboxy methyl ligstroside aglycone (also known as oleocanthal), a phenolic compound contained in EVOO, has been reported to provide *per se* both sensory and anti-inflammatory attributes of EVOO, thus contributing to the health benefits associated with the Mediterranean dietary pattern [43]. Oleocanthal is homologous with the non-steroidal anti-inflammatory drug ibuprofen [44] for both perceptual and anti-inflammatory properties. Therefore, it is tempting to speculate that long term consumption of EVOO containing this compound may contribute to significantly reduce the development and/or the progression of chronic inflammatory diseases and to increase the response to biological agents, thus representing a nutritional marker of responsiveness to Tumor Necrosis Factor-α blockers in psoriasis, in association with genetic marker, such as Interleukin 6 gene promoter polymorphism [45]. In this context, we evaluated also the relationship between the CRP levels, produced by the liver under the influence of IL-6 as one of the links between the altered cytokine milieu and psoriasis, and the consumption of EVOO. Indeed, we found higher consumption of EVOO and lower CRP levels in the control group compared with psoriatic patients.

## Conclusions

This is the first study to evaluate the association between adherence to the Mediterranean diet and the severity of psoriasis, with a strict relationship between a higher consumption of EVOO and a lower psoriasis severity. Although we are aware that the reduced sample size and the validity of BIA in predicting the FM might be a limitation of our study, the association between the Mediterranean Diet and psoriasis severity suggests the possible beneficial effects of nutritional interventions promoting a Mediterranean food pattern rich in EVOO, fruits, vegetables, fish, chicken and whole grains, as an inexpensive and safe adjuvant treatment for psoriatic patients. Moreover, our study highlights the usefulness of the assessment of body composition by BIA in the evaluation of the psoriatic patients. Further researches on larger sample size are needed to unravel the individual role of the nutrients in the diet on the severity of psoriasis, particularly regarding the naturally occurring anti-inflammatory properties of olecanthal. The Nutritionists should play a central role in the evaluation and management of psoriatic patients.

## Abbreviations

EVOO: Extra virgin olive oil
BMI: Body mass index
PASI: Psoriasis area and severity index
CRP: C-reactive protein
WC: Waist circumference
BIA: Bioelectrical impedance analysis
PREDIMED: PREvención con DIeta MEDiterránea
R: Resistance
Xc: Reactance
R/H: Resistance/Height
Xc/H: Reactance/Height
PA: Phase angle
Na/K: Sodium/Potassium
BCMI: Body cell mass index
FM: Fat mass
FFM: Fat-free mass
BCM: Body cell mass
SMM: Skeletal muscle mass
TBW: Total body water
ECW: Extra-cellular water
ICW: Intra-cellular water
FFQ: Food frequency questionnaire
